# A Procedural Approach to Remembering Personal Identification Numbers among Older Adults

**DOI:** 10.1371/journal.pone.0025428

**Published:** 2011-10-05

**Authors:** Michael K. Gardner, Robert D. Hill, Christopher A. Was

**Affiliations:** 1 Department of Educational Psychology, University of Utah, Salt Lake City, Utah, United States of America; 2 Department of Educational Foundations & Special Services, Kent State University, Kent, Ohio, United States of America; University of Minnesota, United States of America

## Abstract

This study investigated whether a motor skill learning intervention could provide better memory for personal identification numbers (PINs) as compared to a control group. Younger (ages 18 to 40) and older (ages 61 to 92) participants were randomly assigned to conditions. All participants received three days of training consisting of 12 blocks of 12 trials each. Participants were tested immediately after training, after four days, and after seven days. Dependent measures were errors, latencies, and number of correct responses per minute. Younger participants were less error prone, faster, and produced more correct responses than older participants. Training condition (motor skill-based versus control training) had no significant effect on any of the dependent variables. Testing time had a significant effect on latency, and the effect of testing time on latency interacted with age group. In a second study, six older individuals diagnosed as having mild cognitive impairment (MCI) were trained using the motor skill learning intervention. Their performance was compared with that of the younger and older motor skill groups from the first experiment. The results showed that the older MCI group was significantly slower, more error prone, and produced fewer correct responses per minute than the older, normal group. Thus the presence of diagnosed MCI significantly impairs memory for PINs beyond the impairment expected from normal aging.

## Introduction

Retrieval of everyday numbers is among the more commonly reported cognitive complaints in older adults [1]. Most current memory interventions for the elderly focus either on verbal material or on name-face recall [2], [3]. However, as society continues its move toward greater reliance on computers and technology, memory for numbers has grown in importance. For instance, personal identification numbers (PINs) are used to control unauthorized access to banking services [4] and credit card accounts. PINs are also used to access social security services via “smart cards” [5]. It has been estimated that the typical homeowner will have PINs for at least three different credit and/or debit card accounts [6]. When other numerically-based security codes are factored in, the number of PINs or PIN-like material to be remembered can be staggering.

Of course, one could solve the problem of memory for PINs by simply writing them down. However, the purpose of PINs is to provide a secure way of identifying account owners or those eligible for services. Security is compromised when writing PINs down; if the list is found by an individual who already knows the account number (or who can easily obtain it), this individual can remove funds, fraudulently obtain services, or otherwise commit identity fraud. For this reason, most institutions encourage the memorization of PINs.

Another possibility would be to set all of one's PINs to a single PIN, thus reducing the problem to that of remembering a single PIN. While appealing, this strategy is usually not feasible. Most PINs are initially set by the institution that provides the account. Many times resetting the PIN to a new number is difficult or impossible. Sometimes resetting the PIN results in the institution providing you with a new random number, rather than a number that you specify. In addition, many institutions require PINs that adhere to a specific set of rules (e.g., must be at least eight digits long, with no more than two of the same digit in succession). Finally, using a single PIN reduces overall account security.

Older adults have a great deal of difficulty remembering numeric material, in part because, unlike verbal stimuli or names and faces, numbers are abstract. Most mnemonic strategies used with older adults treat the to-be-remembered material as declarative knowledge that needs to be consciously encoded and then recalled [3]. This is true for both verbal material and numeric material [7], [8], [9]. However, the abstract nature of numbers makes them poor candidates for declarative mnemonic strategies that are commonly based upon verbal association, visualization, or elaboration, all of which are deficit in older adults. What is needed is a strategy that places fewer demands upon declarative processes.

We believe it possible to teach individuals PINs by treating the problem as one of acquiring procedural knowledge: that of entering the PIN into a keypad. There is ample evidence from work in experimental psychology that procedural memory (sometimes referred to as “implicit memory”) operates in different ways than declarative memory (or “explicit memory” [10]). Further, implicit processes for retrieval of information are subject to different limitations [11], [12], [13], but also have certain advantages, including the potential for memory consolidation of declarative knowledge (in the form of rules or sequences of operations) with minimal demand on explicit memory [14]. There is also evidence that implicit memory does not decline with aging [15]. Castel [16] found that individuals who routinely manipulate numbers display good memory for them, and retain this ability into old age. Thus, there is reason to believe that a procedural approach for learning and later recall of numbers might succeed where declaratively-based mnemonic approaches have failed. The procedural approach evaluated in this study consisted of teaching individuals the motor skill of entering each PIN into a keypad in response to appropriate contextual cues (i.e., the particular setting where PIN entry is required).

In designing our procedural memory training intervention, we followed principles developed by Glisky and her colleagues [17], [18], [19], [20], [21], [22] and Baddeley and his colleagues [23], [24]. Glisky developed a technique called the method of vanishing cues for teaching amnesic individuals declarative knowledge. The procedure involved presenting participants with sufficient “cueing information” so that they could respond correctly to a set of memory items. The cueing information was then reduced over subsequent occurrences of the items. By the end of training, participants could respond correctly with only minimal cueing. This technique is similar to the behavioral principle of “fading”, whereby contingencies for a desired behavior are gradually reduced once the behavior becomes an established part of the individual's repertoire [25].

Another essential component of the current procedural training intervention was the minimization of errors during training. Baddeley has shown the importance of errorless learning, especially with regard to learning in amnesiacs. Wilson et al. [24] demonstrated that a condition that required amnesiacs to produce guesses (which were usually errors) on memory items resulted in poorer learning than a condition that prevented guessing. Presumably this finding occurs because in the guessing condition the errors become incorporated into the mental representation of the knowledge, while this does not happen in the error free condition. This demonstrates that any application of the method of vanishing cues must be sensitive to the issue of errors during acquisition, and must reduce these errors as much as possible.

In this paper we present a procedurally-based, motor skill training program designed to teach individuals numeric material: namely, PINs for later recall. We compared the performance of this approach to a conventional approach of simply trying to remember the PINs (which presumably involves treating the numbers as declarative knowledge stimuli to be consciously retrieved). We also compare the performance of older participants to that of younger participants. In a second study we trained a small number of older individuals (i.e., six) who were diagnosed with pre-Alzheimer's mild cognitive impairment (MCI) using the same procedurally-based, motor skill program. We then compared their performance with that of the non-impaired older and younger motor skills training groups from the first experiment.

## Experiment I

### Methods

#### Participants

Older healthy participants were recruited from agencies within the Salt Lake City area that had contact with older adults. Salt Lake County Aging Services allowed us to recruit participants from programs they sponsored, as well as via their network of 18 Senior Centers that provide activities and meals to older adults. Participants recruited through Salt Lake County Aging Services were paid $25 for their participation. Participants recruited from these source were consented using a standard written, non-HIPPA, consent form (available upon request), and this aspect of the study was approved by the University of Utah Institutional Review Board as IRB_00012721 entitled “A Motor-Skill Approach to Remembering Personal Identification Numbers in the Elderly”. The University of Utah's Center for Alzheimer's Care, Imaging & Research allowed us to recruit healthy older adults from their list of potential research participants (as well as older adults diagnosed with mild cognitive impairment [MCI], whose results are reported in Experiment II). Participants recruited through the Center for Alzheimer's Care, Imaging & Research were paid $75 for their participation. Participants recruited from the Center for Alzheimer's Care, Imaging & Research were consented using a written HIPPA consent form (available upon request), and this aspect of the study was approved by the University of Utah Institutional Review Board as IRB_00031279 entitled “Motor-Skills Training of PIN in the Elderly With and Without MCI”. Older participants were screened for depression and adequate mental status (see Procedures: Overall Procedures for a full description of the screening measures).

Younger participants were recruited via the participant pool in the Department of Educational Psychology at the University of Utah. Individuals in this pool are taking one of a number of undergraduate courses in Educational Psychology that contain a research component. As part of these courses, students participate in approved research projects (other ways of completing the requirement also exist). These participants were screened to ensure that their ages were between 18 and 40 years. These participants were consented using a standard, non-HIPPA, consent form (available upon request), and this aspect of the study was approved by the University of Utah Institutional Review Board as IRB_00012721 entitled “A Motor-Skill Approach to Remembering Personal Identification Numbers in the Elderly”.

A total of 55 participants between the ages of 61 and 92 were recruited and randomly assigned to either the procedurally-based, motor skills condition (N = 29) or the control condition (N = 26). The older procedural group consisted of 19 females and 10 males. The average age for this group was 72.34, with a standard deviation 7.97. The average education (in years) was 15.17, with a standard deviation of 2.44. The older control group consisted of 19 females and 7 males. The average age for this group was 71.31, with a standard deviation of 7.64. The average education (in years) was 15.35, with a standard deviation of 2.87. A total of 37 participants between the ages of 18 and 40 were recruited. These were randomly assigned to either the procedural-based, motor skills condition (N = 22) or the control condition (N = 15). The younger procedural group consisted of 16 females and 6 males. The average age for this group was 22.14, with a standard deviation 3.30. The average education (in years) was 15.09, with a standard deviation of 1.66. The younger control group consisted of 8 females and 7 males. The average age for this group was 24.67, with a standard deviation of 4.92. The average education (in years) was 15.07, with a standard deviation of 2.28. A two-way analysis of variance (age group by training condition) confirmed that only difference was between the groups was age (for age group, *F*[1,88]  = 1171.87, *p*<.001). There were no other significant differences between the groups on age or education.

#### Overall procedures

Participants completed five sessions spaced over a two week period. The sessions were scheduled on Monday, Wednesday, and Friday of the first week, and on Tuesday and Friday of the second week. Procedural, motor skills training, or the corresponding control training, took place during the first three sessions. Memory testing took place immediately after the last training session (i.e., Friday) and on each of the last two sessions (i.e., the following Tuesday and Friday).

Other tasks also occurred across the five sessions. During session one, participants gave informed consent, filled out a brief demographic questionnaire (which asked for participant gender, age, years of education, and whether or not the participants had any physical or emotional condition that would interfere with his or her ability to enter information at a computer keypad), completed the Short Portable Mental Status Questionnaire [26] and the Geriatric Depression Scale [27]. The latter two instruments served as screening measures for cognitive impairment and depression. Participants were excluded if they made four or more errors on the Short Portable Mental Status Questionnaire or scored five or higher on the Geriatric Depression Scale. Two participants were excluded due to performance on the Short Portable Mental Status Questionnaire, and four were excluded due to performance on the Geriatric Depression Scale. The demographic data reported earlier represents only those participants who passed all screening measures.

#### Procedural Motor Skills Training: Structure of a Trial

Participants in this study were attempting to learn four four-digit PINs, and to correctly associate each PIN with its context of usage (i.e., bank, telephone, grocery, and computer). On each training trial, the following sequence of events transpired: (a) a blank screen was presented for 500 msec; (b) the name of one of the four contexts appeared for 1000 msec; (c) a screen appeared with pictures of all four contexts; (d) the participant responded by pressing an arrow key on the keyboard that indicated the position of the picture that matched the previously presented context (e.g., bank); (e) if the participant pressed an incorrect arrow key, auditory feedback was presented (i.e., “incorrect”) and the same trial re-initiated from the beginning, otherwise the trial continued to the next step; (f) a blank screen was presented for 500 msec; (g) some of the PIN number was presented as “cueing” information” (how many digits were presented varied over blocks of trials, with less information being presented in later blocks; during blocks 1 and 2, all four digits were present; during blocks 3 and 4, the first three digits were presented; during blocks 5 and 6, the first two digits were presented; during blocks 7 and 8, the first digit was presented; and during blocks 9 through 12, only a blank screen was presented); (h) in the presence of the cueing information, the participant entered the entire four-digit PIN into a separate USB keypad with their right hand; (i) if the participant's response was correct, the trial ended; if the participant's response was incorrect, the participant received auditory feedback (i.e., “incorrect”) and was shown the correct PIN until he or she pressed any key on the keyboard, which removed the feedback; once the feedback terminated, the same trial re-initiated from the very beginning.

The important aspects of the procedural, motor skills trials were: (a) requiring the participant to not only remember the PIN, but associate it with the correct context of usage; (b) learning was supported by substantial cueing information that was systematically reduced, using the method of vanishing cues, over blocks of trials; (c) errors were minimized by requiring participants to respond correctly before they could continue on to the next trial; and (d) the training emphasized the importance of motor sequencing in acquiring of the skill of entering the PIN at the keypad, rather than conscious recall of the digit sequence verbally.

#### Control Training: Structure of a Trial

The control training was designed to be parallel to the procedural, motor skills training, but to leave participants to their own strategy to decide how to remember the PINs. This presumably would encourage participants to remember the PINs as pieces of declarative knowledge. The only prohibition was that participants were not allowed to enter the PINs into the keypad. This was done to provide as clear a distinction between the two conditions as possible.

On each training trial, the following sequence of events transpired: (a) a blank screen was presented for 500 msec; (b) the name of one of the four contexts appeared for 1000 msec; (c) a screen appeared with pictures of all four contexts along with an arrow pointing to the correct picture; (d) the participant studied this for as long as needed, with the following constraints: the participant was forced to view this information for at least 1000 msec, after which time a message appeared that said “You may now press the spacebar to move on”; and if the participant did not respond with a spacebar press by 10000 msec a message appeared saying “Your time is up” and the trial continued (participants were informed of the 10000 msec time limit in the instructions); (e) the entire PIN number was presented for the participant to study, with the following constraints: the participant was forced to view this information for at least 1000 msec, after which time a message appeared that said “You may now press the spacebar to move on”; and if the participant did not respond with a spacebar press by 10000 msec a message appeared saying “Your time is up” and the trial terminated (participants were informed of the 10000 msec time limit in the instructions).

Participants in the control condition: (a) were instructed to use whatever strategy they could devise to remember the PINs (which, presumably, would be a declaratively-based strategy such as rehearsal); (b) saw the entire PIN on all trials (no method of vanishing cues was used); (c) could not technically make errors, because the PIN was not required as a response; and (d) could not develop a motor procedure for PIN entry, because the PIN was never entered into a keypad.

#### Testing Sessions: Both Conditions

Each testing session consisted of a single trial for each of the four PINs. The participant was shown the picture and name of the location for each PIN (the order was randomized over participants). He or she then entered the PIN for that location into the USB keypad. Response time and correctness of response were recorded for each digit at each serial position for each PIN. Testing sessions and training sessions were programmed using E-Prime [28], an authoring system for developing computerized experiments.

### Results

#### Training Data: Latency

Participants in the procedural, motor-skill condition were trained to acquire a skill. Latencies to acquire a skill should follow the power law of learning [29]. A power function was fitted to participants' group training data (for each training session) as a function of age group (old versus young) and learning condition (experimental [i.e., procedural, motor skill condition] versus control [i.e., no explicit strategy given]). These data are displayed in Table 1. The form of the function fit was RT  =  a(T)^b^, where RT is response time latency (in msec), a is the power function constant, T is the trial number within session, and b is the power function slope or exponent. Table 1 presents model fit (i.e., multiple R^2^), power function slope, and power function constant for old and young participants as a function of training condition (procedural motor skill versus control) and training session (one through three). As can be seen from the Table, participants training latencies were well fit by the power function in the procedural, motor-skill groups for both younger and older groups. The fit decreased over sessions as participants began to approach asymptote. For the controls groups (both younger and older) there was a good fit during session 1, but poor fits thereafter. We interpret this pattern as follows: both learning conditions were acquiring the skill of participating in the training phase of the study, thus both learning conditions showed a power function during the first training session. However, the procedural, motor-skills groups were also acquiring the motor skill of entering the PINs into the keypad. This skill took much longer to acquire, and thus their data showed a power function fit during sessions 2 and 3, while the control groups did not show this pattern. The overall pattern of latencies during training supports the notion that the motor-skills groups were, indeed, acquiring a procedural skill for entering the PINs into the keypad.

**Table 1:** Power Function Fits (Multiple R^2^) and Unstandardized Power Parameters to Training Latency Data as a Function of Age Group, Learning Condition and Training Session.

Age Group	Learning Condition	Parameter	Session 1	Session 2	Session 3
Young					
	Motor Skill Group				
		Multiple R Square	0.928	0.691	0.327
		Power Slope	−0.351	−0.125	−0.045
		Power Constant	4398	2330	1965
	Control Group				
		Multiple R Square	0.878	0.216	0.031
		Power Slope	−0.541	−0.044	−0.023
		Power Constant	2346	657	612
Old					
	Motor Skill Group				
		Multiple R Square	0.974	0.840	0.752
		Power Slope	−0.372	−0.162	−0.127
		Power Constant	5133	2677	2001
	Control Group				
		Multiple R Square	0.950	0.018	0.463
		Power Slope	−0.613	0.028	−0.079
		Power Constant	4902	1404	1750

#### Testing Data: Error Rate

**Figure 1:**
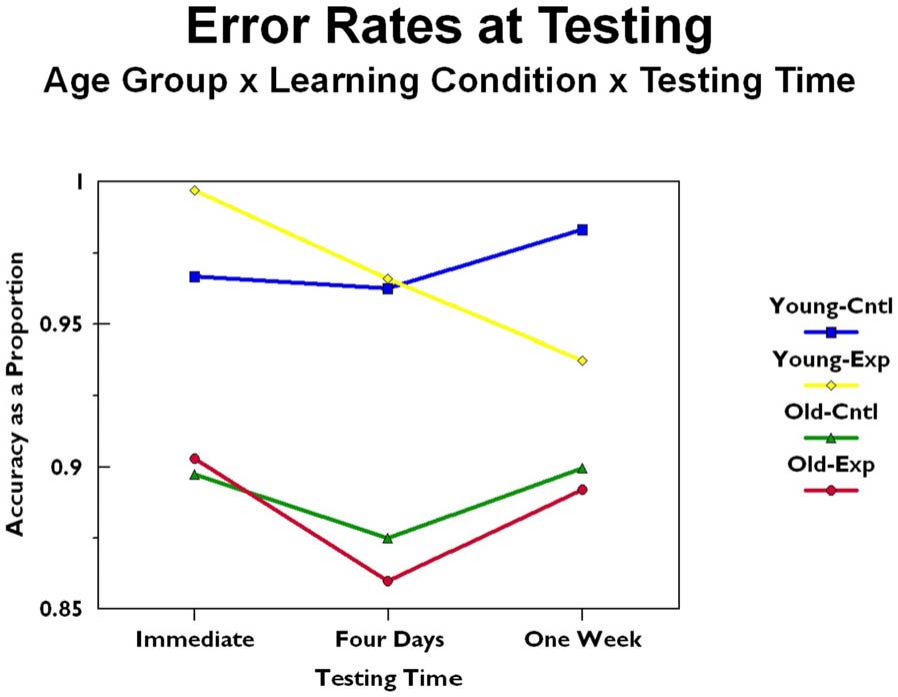
Error rates during testing as a function of age group, training condition, and testing time.

Perhaps the most straightforward measure of participant performance is error rate. This was calculating at each testing time by summing the number of digits correctly recalled (in their correct serial position) and dividing by 16 (the total number correct that was possible). This yielded error rate as a proportion. These data are presented in Figure 1 as a function of age group (old versus young) and learning condition (experimental [i.e., procedural, motor skill condition] versus control [i.e., no explicit strategy given]), and testing time (immediate, four days after training, and seven days after training). The error rates were analyzed using a three factor mixed model analysis of variance with age group and learning condition as between subject factors and testing time as a repeated measures factor. There was a significant main effect for age (*F*[1, 90]  = 8.735, *p*<.01), with younger participants consistently displaying more accurate memory than older participants (for young: immediate *M* = 0.9848, four day *M* = 0.9645, seven day *M* = 0.9561; for old: immediate *M* = 0.9163, four day *M* = 0.8728, seven day *M* = 0.9007). Procedural, motor skills training was not significantly better than the control condition (*F* [1,90]  = 0.030, *p*>.10). No other main effects or interactions were significant.

#### Testing Data: Latency

One problem with error rate as a dependent variable is that it has limited variability. On each serial position of each PIN, a participant can only achieve one of two outcomes: either the digit was correct or it was incorrect. Latency has the benefit of constituting a more sensitive dependent variable. Thus, latency might reveal trends in the data that accuracy masks (for measurement related reasons).

**Figure 2:**
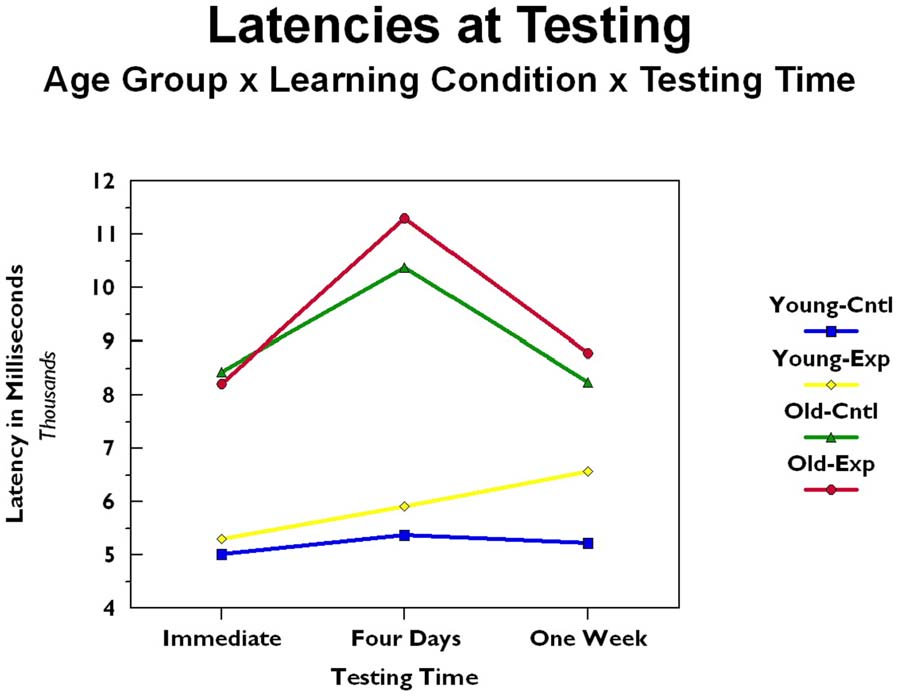
Latencies during testing as a function of age group, training condition, and testing time.

For each participant, latency, in milliseconds, was recorded for each entry of each digit of each PIN. These latencies were for all responses, including errors. Overall latency was calculated as the average latency for PINs for each participant (i.e., for each participant and each PIN, the latencies for the four digits were summed to get a total PIN latency; these PIN latencies were averaged to get a participant latency per PIN). These data are presented in Figure 2 as a function of age group (old versus young) and learning condition (experimental [i.e., procedural, motor skill condition] versus control [i.e., no explicit strategy given]), and testing time (immediate, four days after training, and seven days after training). The latencies were analyzed using a three factor mixed model analysis of variance with age group and learning condition being between subject factors and testing time being a repeated measures factor. As with error rate, there was a significant main effect of age (*F*[1, 90]  = 19.926, *p*<.001). Younger participants were consistently faster than older participants (for young: immediate *M* = 5196 msec, four day *M* = 5701 msec, seven day *M* = 6029 msec; for old: immediate *M* = 8320 msec, four day *M* = 10841 msec, seven day *M* = 8495 msec). In addition, the main effect of testing time was significant (*F*[2, 180]  = 5.986, *p*<.01; immediate *M* = 7077 msec; four day *M* = 8796 msec; seven day *M* = 7514 msec), as was the interaction between testing time and age (*F*[2, 180]  =  4.631, *p* = .01; see Figure 2). These effects were due to an increase in latency at four day testing, primarily occurring in the older age group. It seems that older participants were relatively fast when testing immediately followed a lengthy training session during which they had time to become accustomed to the task. However, when tested four days later without training, older participants' response times slowed. Upon return at seven days after training, the older participants were better able to anticipate the testing task, demonstrated by faster response times (as compared to four day testing). Younger participants were relatively fast in responding at all testing times. No other effects were significant.

#### Testing Data: Speed

While latency has greater variability than error rate data, both latency and error rates suffer from being non-normal distributions. One potential solution has been suggested by Woltz [30], [31], [32]: convert the latency and error rates into a composite measure of speed per correct response. This can be done by taking error rate and dividing it by latency in minutes (which is latency in msec divided by 60000) yielding the number of correct responses per minute or a measure of speed of responding. This measure has a greater tendency toward normality, and incorporates aspects of both error rate data and latency data. Thus effects that take into account variance in both dependent variables are unified in this new variable.

**Figure 3:**
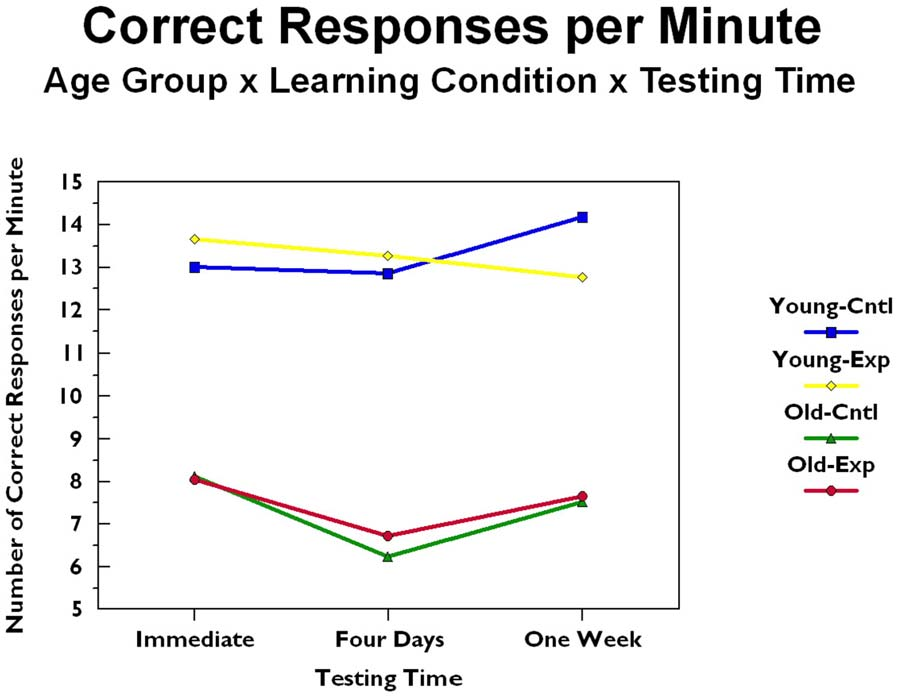
Speed during testing as a function of age group, training condition, and testing time.

This speed measure is depicted in Figure 3 as a function of age group (old versus young) and learning condition (experimental [i.e., procedural, motor skill condition] versus control [i.e., no explicit strategy given]), and testing time (immediate, four days after training, and seven days after training). A three factor mixed model analysis of variance with age group and learning condition as the between subject factors and testing time as the repeated measures factor. The main effect of age group was significant (*F*[1. 90]  = 44.664, *p*<.001), with younger participants producing more correct responses per minute than older participants (young, *M* = 13.29; old, *M* = 7.39). The main effect of testing time approached significance (*F*[2, 180 = 2.550, *p* = .08; testing time: immediate *M* = 10.18, four day *M* = 9.10, seven day *M* = 9.85), presumably due to the increase in latencies found among the older group at the four day testing time. This increase in latencies had the effect of decreasing the number of correct responses per minute at that testing time. No other effects were significant.

#### Ability Measures

We measured participants' verbal ability and reasoning ability using the Shipley Vocabulary Scale [33] and the Shipley Inference Scale [33]. Participants completed the Shipley Vocabulary Scale on Tuesday of the second week of testing after completing the four-day PIN recall task, and completed the Shipley Inference Scale on Friday of the second week of testing after completing the seven-day PIN recall task. Means and standard deviations for these measures (as a function of age group and learning condition are presented in Table 2.

**Table 2:** Means and Standard Deviations for the Shipley Vocabulary Scale and Shipley Inference Scale as a Function of Age Group, Learning Condition.

Age Group	Learning Condition	Parameter	Vocabulary	Inference
Young				
	Motor-Skill Group			
		Mean	29.05	16.50
		Standard Deviation	5.028	2.087
	Control Group			
		Mean	27.93	16.40
		Standard Deviation	4.636	2.501
Old				
	Motor-Skill Group			
		Mean	32.66	14.21
		Standard Deviation	7.330	4.701
	Control Group			
		Mean	31.08	13.76
		Standard Deviation	6.633	3.345

Both measures were analyzed using a two-way analysis of variance, with the factors being age group (old versus young) and learning condition (experimental [i.e., procedural, motor skill condition] versus control [i.e., no explicit strategy given]). Due to missing data for one individual on the Shipley Inference Scale, the degrees of freedom do not match for the two analyses. For the Shipley Vocabulary Scale, there was a main effect of age group, with older participants scoring higher than younger participants (*F*[1,88]  = 6.311, p<.05; old: *M* = 31.91; young: *M* = 28.60). None of the other effects were significant. For the Shipley Inference Scale, there was again a significant main effect for age, with younger participants scoring higher than older participants (*F*[1,87]  = 10.639, p<.01; old: *M* = 14.00; young: *M* = 16.49). None of the other effects were significant. This pattern of better performance on fluid measures (e.g., Shipley Inference) by younger individuals, but better performance on crystallized measures (e.g., Shipley Vocabulary) by older individuals, is consistent with Horn and Cattell's theory of fluid and crystallized intelligence [34], [35], [36], [37], [38], [39], [40] (a description of the theory is available by Gardner [41]).

Correlations were calculated between the Shipley Vocabulary and Shipley Inference scores and each individual's error rate, latency, and speed (averaged across all three testing times). These correlations were calculated within each combination of age group and learning condition to determine if the relationships changed as a function of these categorical variables.

For older participants in the procedural, motor skill condition there was a significant relationship between Shipley Inference and average latency (*r* [29]  = −0.52, *p*<.01) and between Shipley Inference and average speed (*r* [29]  = 0.45, *p*<.05). It should be pointed out that latency and speed are not experimentally independent, since speed is derived from latency and error rate. Thus, the correlation primarily reflects the fact that those with higher fluid ability scores had lower latencies in responding during testing. Both of these variables may be indicative of a higher level of overall mental functioning.

For older participants in the control condition, none of the relationships between the Shipley Vocabulary and Shipley Inference with error rate, latency and speed were significant. This was also true for both groups of younger participants (procedural, motor skill condition and control condition): there were no significant correlations between either of the Shipley measures and any of the dependent measures. In considering these results, it should be pointed out that in some combinations of age group and learning condition the number of participants was quite small (as low as 15). Thus, the lack of significance of these correlations is far from conclusive.

## Experiment II

Experiment II represented a pilot study utilizing a small (N = 6) group of individuals who had been diagnosed, using standard protocols, with pre-Alzheimer's MCI. These individuals were trained using the same procedurally-based motor skills program described in Experiment I, and were tested in the same fashion as in Experiment I.

### Method

#### Participants

The participants in Experiment II were six older individuals diagnosed with pre-Alzheimer's mild cognitive impairment by University of Utah's Center for Alzheimer's Care, Imaging & Research. These individuals had volunteered to participate in research projects, and were paid $75 for their participation. They were consented using a written HIPPA consent form (available upon request), and this aspect of the study was approved by the University of Utah Institutional Review Board as IRB_00031279 entitled “Motor-Skills Training of PIN in the Elderly With and Without MCI”. Older participants were screened for depression and adequate mental status in the same manner described above for healthy participants.

The MCI group consisted of 2 females and 4 males. This group had an average age of 78.00 years, with a standard deviation of 5.69. The average education (in years) was 16.67, with a standard deviation of 3.88. Shipley Vocabulary and Inference scores were available for 5 of the 6 participants (one participant's Shipley data was lost due to a computer malfunction). The average Shipley Vocabulary Scale score was 34.20, with a standard deviation of 2.683. The average Shipley Inference Scale score was 10.80, with a standard deviation of 2.049.

#### Procedures

All MCI participants were trained in the procedurally-based, motor skills training program described in Experiment I. All training and testing procedures were identical to Experiment I.

## Results

The MCI group's accuracy, latency, and speed data were collected as described in Experiment I. For purposes of comparison, we conducted two-way ANOVAs comparing the younger, normal older, and MCI older groups across the three testing times (immediate, four day, and seven day). All of these groups had been trained using the procedurally-based, motor skill procedure (i.e., no control groups are included, as we did not have an MCI control group due to the difficulty of recruiting MCI participants for a time intensive study with no active treatment).

### 

#### Testing Data: Error Rate

The main effect of participant group on accuracy was significant, *F*(2,54)  = 13.989, *p*<.001. A Tukey HSD post-hoc test revealed that both the old and young normal groups, which did not differ from each other, were more accurate than the MCI group (*p*'s<.001). The main effect of testing time was also significant for accuracy, *F*(2,108)  = 3.385, *p*<.05. This was due to a decline in accuracy for testing at four and seven days post training, as compared with testing immediately following training. The interaction of participant group and testing time was not significant.

#### Testing Data: Latency

The main effect of participant group on latency was significant, *F*(2,54)  = 15.668, *p*<.001. A Tukey HSD post-hoc test revealed that both the old and young normal groups, which did not differ from each other, were faster in responding than the MCI group. The main effect of testing time was also significant for latency, *F*(2,108)  = 6.076, *p*<.01. This reflected a general increase in latency from immediate testing, through four day testing, and seven day testing. The interaction of participant group and testing time was also significant, *F*(4,108)  = 2.769, *p*<.05. This was due to the fact that both the young normal group and the MCI group showed a monotonic increase in latency with increased delay. However, the old normal group showed an increase in latency from immediate testing to four day testing, but a decrease in latency from four day testing to seven day testing. MCI participants saw an increase in latency at greater testing delays, and this was much larger in magnitude than that experienced by younger participants.

#### Testing Data: Speed

The speed transformation combined accuracy and latency into a single variable: number of correct responses per minute. The main effect of participant group on speed was significant, *F*(2,54)  = 125.533, *p*<.001. A Tukey HSD post-hoc test revealed that all three groups differed (old normal vs. young normal, *p*<.001; old normal vs. MCI, *p*<.05; young normal vs. MCI, *p*<.001). The order of condition was: young normal participants responded fastest, followed by the old normal participants, and finally the MCI participants. The main effect of testing time, and the interaction of testing time and participant group, were not significant.

The pilot study involved six individuals with diagnosed MCI. These participants were indeed impaired with respect to their ability to remember PINs: their performance was significantly below that of normal, healthy older individuals. However, the intervention did not seem to provide noticeable benefits.

## Discussion

Despite claims by some researchers that procedural memory is supported by different memory systems than declarative memory (e.g., Squire [42], [43]), and that learning may be possible in the procedural domain even when declarative memory processes are impaired (e.g., Glisky and Schacter [20]), this may not be the case for older adults (or, at least, it may not be true in general for older adults). Perhaps procedural memory processes show deficits with age, and are subject to impairment by certain type of organic damage (such as that suffered by our MCI group). Our results are consistent with this possibility; however, whether a procedural training paradigm can be employed to offset age-related or disease-mediated memory deficits for everyday numbers remains an open question.

It may be that the current study did not provide an adequate test of procedural learning processes. For instance, although both younger and older individuals in the control group learned 4 four-digit PINs using declarative memory when given the lengthy training exposure provided in the current study, there were no additional advantages when using the procedurally-based motor skills training. Perhaps a better test would have been to increase the number of PINs to eight or sixteen. This might have created greater degree of overload for declarative memory, and resulted in greater differences between the experimental and control conditions. While we did consider increasing the number of PINs in the current study, we weighed the advantages it would afford against the possibility that such an approach would be challenging for healthy older adults, and potentially impossible for persons with MCI.

The necessity for robust training strategies to support numeric memory, especially among the elderly, is large and likely to continue to grow in the future. Our study examined the possibility of taking declarative information (the 4 four-digit PINs) and converting it to procedural knowledge (the act of entering the PINs at a keypad in the presence of appropriate environmental stimulus cues). While not successful, we are equally dubious that mnemonic techniques that require the individual to overlearn a complicated system (e.g., the number-consonant mnemonic) will be successful with the average elderly population. We believe that there must be ways to support numeric memory in average older adults. The positive impact a successful technique would have cannot be overestimated.
